# Statistical Inference of Selection and Divergence of the Rice Blast Resistance Gene *Pi-ta*

**DOI:** 10.1534/g3.114.014969

**Published:** 2014-10-21

**Authors:** Amei Amei, Seonghee Lee, Kirankumar S. Mysore, Yulin Jia

**Affiliations:** * Department of Mathematical Sciences, University of Nevada, Las Vegas, Nevada; † Plant Biology Division, The Samuel Roberts Noble Foundation, Ardmore, Oklahoma; ‡ USDA-Agricultural Research Service, Dale Bumpers National Rice Research Center, Stuttgart, Arkansas

**Keywords:** evolution, NBS-LRR, *Pi-ta*, resistance gene, Poisson random field

## Abstract

The resistance gene *Pi-ta* has been effectively used to control rice blast disease, but some populations of cultivated and wild rice have evolved resistance. Insights into the evolutionary processes that led to this resistance during crop domestication may be inferred from the population history of domesticated and wild rice strains. In this study, we applied a recently developed statistical method, time-dependent Poisson random field model, to examine the evolution of the *Pi-ta* gene in cultivated and weedy rice. Our study suggests that the *Pi-ta* gene may have more recently introgressed into cultivated rice, *indica* and *japonica*, and *U.S. weedy rice* from the wild species, *O. rufipogon*. In addition, the *Pi-ta* gene is under positive selection in *japonica*, *tropical japonica*, *U.S. cultivars* and *U.S. weedy rice*. We also found that sequences of two domains of the *Pi-ta* gene, the nucleotide binding site and leucine-rich repeat domain, are highly conserved among all rice accessions examined. Our results provide a valuable analytical tool for understanding the evolution of disease resistance genes in crop plants.

Blast disease, caused by the filamentous fungus *Magnaporthe oryzae*, is one of the most damaging diseases of rice. Genetic analysis has identified more than 100 major and minor resistance (*R*) genes (Ashikawa *et al.* 2008). Thus far, 22 of these *R* genes have been cloned, and most of the genes encode putative receptor proteins with nucleotide binding sites (NBS) and leucine-rich repeat (LRR) domains (Lei *et al.* 2013). The presence of NBS and LRR domains in *R* genes suggests that they are involved in signal recognition, protein−protein interaction, and ligand binding for initiating plant disease responses (DeYoung and Innes 2006). One of the best-studied *R* genes for blast disease (Bryan *et al.* 2000) is *Pi-ta*, which encodes a putative protein with NBS and degenerate LRR domains. *Pi-ta* has been used effectively in preventing infection by strains of *M. oryzae* in rice (Bryan *et al.* 2000; Orbach *et al.* 2000). Baseline information on the structure, function, natural variation of the *Pi-ta* allele, and signal recognition of *Pi-ta* has accumulated from a concerted effort to understand the factors governing rice disease (Jia *et al.* 2000, 2003; Wang *et al.* 2008). All the resulting knowledge to understand the molecular evolution of the *Pi-ta* gene has been used effectively for developing disease-resistant varieties against rice blast (Jia 2003; Jia *et al.* 2002, 2004a).

The resistance *Pi-ta* allele, isolated from landrace varieties Tetep (Vietnam) and Taducan (Philippine), was introgressed into a diverse range of rice varieties to prevent rice blast (Wang *et al.* 2007). However, strains carrying the resistance *Pi-ta* contain a large linkage block of 5.3 Mb that is difficult to break, even after five generations of backcrossing under selection for blast resistance (Jia 2009; Jia *et al.* 2009b). Consequently, it has been a real challenge to clone additional components involved in the *Pi-ta*−mediated resistance pathway (Jia and Martin 2008). Sequence analysis of genomic regions surrounding the *Pi-ta* gene in 159 geographically diverse accessions of *O. sativa*, *O. rufipogon*, and five other closely related AA genome *Oryza* species, *O. nivara*, *O. meridionalis*, *O. glaberrima*, *O. barthii*, and *O. glumaepatula*, demonstrated that a single amino acid change determines resistance specificity (Bryan *et al.* 2000; Orbach *et al.* 2000; Lee *et al.* 2009)

The *R* genes encoding NBS-LRR proteins are the most diversified gene families in dicot and monocot plant species, and the genes have been under strong selection (Huang *et al.* 2008; Thakur *et al.* 2013; Lee *et al.* 2009, 2011). There are about 150 NBS-LRR genes in Arabidopsis and about 400 in rice alone (Mchale *et al.* 2006). When its pathogen successfully defeats the targeted *R* gene, plants are subsequently compelled to evolve a much stronger defense mechanism to survive. It has been known that the pathogens containing high levels of polymorphic effectors trigger the rapid evolution of the alleles with newly arisen mutations of their corresponding *R* genes (Dodds *et al.* 2006; Jia *et al.* 2009b). For example, *R* genes against rice blast are tremendously diverse and evolve rapidly, counteracting the *Avr*-genes in the blast pathogens (Dai *et al.* 2010b; Huang *et al.* 2014). Recent studies have made progress in determining the evolution of the rice blast *R* gene *Pi-ta* and *Pi2/9* locus, and their results have been effectively adopted (Dai *et al.* 2010a; Huang *et al.* 2008; Lee *et al.* 2009, 2011; Zhou *et al.* 2007).

Asian cultivated rice, *Oryza sativa* L., is divided into several genetically distinct groups, including *indica*, *aus*, *aromatic*, *temperate*, and *tropical japonica* (Garris *et al.* 2005). Weedy strains of rice are predicted to possess advantages in natural ecosystems and have been predicted to be either undomesticated or imported (Londo *et al.* 2006; Londo and Schaal 2007). It would be of great interest to determine whether any footprint of the blast *R* genes can be identified among these cultivated rice groups and weedy rice.

In this study, we modeled the evolutionary history of the *Pi-ta* gene by estimating population genetic parameters for 10 rice strains. The objectives of the present study were to i) estimate divergence time of the *Pi-ta* gene in 10 rice groups from their wild-type ancestor *O. rufipogon*; ii) estimate synonymous and nonsynonymous substitution rates of the *Pi-ta* gene across the 10 rice groups; and iii) calculate the magnitude and direction of selection on newly arisen nonsynonymous substitution in the *Pi-ta* gene.

## Materials and Methods

### Data analysis

**Figure 1:**
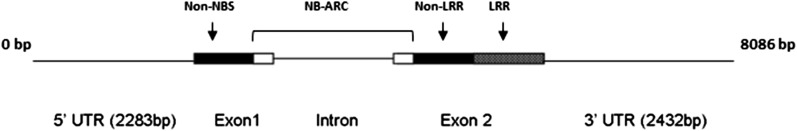
Description of the *Pi-ta* gene structure and domains, non-nucleotide binding site (NBS), NB-ARC, non-leucine-rich repeat (LRR), and LRR. Nucleotides of intron are not included for the region of NB-ARC. All domains contain only coding region sequences of *Pi-ta*.

To compare *Oryza sativa* with its wild relative *O. rufipogon*, a total of 10 artificial groups were assembled. The dataset used in this study contains the full-length coding region (2787 bp) of the *Pi-ta* gene from 10 groups within *O. sativa* [all cultivated *sativa* (57 accessions), *aromatic* (3), *aus* (7), *indica* (22), *japonica* (32), *temperate japonica* (4), *tropical japonica* (13), *U.S. cultivars* (16), weedy rice black hull (BHA) (24) and all weedy rice (58)] and the wild progenitor species *O. rufigopon*. The sequences were obtained from previous studies (Lee *et al.* 2009, 2011). Ten comparison groups were formed by pairing the wild rice species *O. rufigopon* with the 10 rice groups, respectively. Information for the samples is shown in the Supporting Information, File S1, and McDonald-Kreitman tables (McConald and Kreitman 1991) for the 10 rice groups were generated using the software DnaSP 4.9 (Rozas *et al.* 2003). As shown in Figure 1, the *Pi-ta* gene was divided into four functional regions, NB-ARC (APAF-1, R proteins, and CED-4), non-NBS, LRR, and non-LRR, to examine their selective effects individually.

### Model

We used a Markov chain Monte Carlo simulation method to estimate divergence times of the 10 rice pairs, selection coefficients of nonsynonymous substitutions, and two types of substitution rates by applying a time-dependent Poisson random field (PRF) model to the available data (Amei and Sawyer 2010, 2012). Due to the fact that the model is suitable for multiple genes, we treated the four functional regions of a single *Pi-ta* gene as four separate genes under the assumption that nucleotide sites on a gene evolve independently. The traditional McDonald-Kreitman table is a 2 × 2 contingency table consisting of numbers of fixed differences and polymorphisms at synonymous and non-synonymous nucleotide sites. In the time-dependent PRF model, the table has been extended to a 2 × 3 table by classifying polymorphic sites into the following two types: sites that are polymorphic in only one sample and sites that are polymorphic in both samples. Suppose that two closely related species have the same haploid effective population size $N_{e}$; they diverged $t_{div}N_{e}$ generations ago; the synonymous substitution rate of sites in a coding gene in the two lineages is $\theta_{s}$ per site per $N_{e}$ generations ($\theta_{r}$ for nonsynonymous substitution rate); and the selection coefficient of nonsynonymous substitution is *γ* per gene per$N_{e}$ generations. Selective effects of synonymous substitution are assumed to be neutral, *i.e.*, γ=0. Under certain assumptions, such as equal fitness for nonsynonymous substitution on the same locus, constant population sizes, random mating, no migration between species, and genic selection, the six counts in the extended McDonald-Kreitman table are independent Poisson random variables whose means are functions of the genetic parameters $t_{div}$, $\theta_{s}$, $\theta_{r}$ and *γ* (Amei and Sawyer 2010, 2012).

To estimate the parameters of interest using a Bayesian framework, we assume that selection coefficients for the four functional regions, $\gamma_{i}$, 1≤i≤4, are random draws from a normal distribution with mean $\mu_{\gamma }$ and variance $\sigma^{2}$, and an inverse-gamma-normal distribution was assigned as a joint prior distribution of $\mu_{\gamma }$ and $\sigma^{2}$. Gamma distributions with given parameters are chosen as prior distributions for the two types of substitution rates, $\theta_{s}$ and $\theta_{r}$, and a uniform prior distribution for the divergence time $t_{div}$. The posterior distributions of the genetic parameters, given the observed numbers of fixed differences and polymorphisms at synonymous and nonsynonymous sites, are obtained by Markov chain Monte Carlo simulations (see detailed expression of the likelihood function in Amei and Sawyer 2012).

In our model, the $\theta_{s}$ represents an aggregated synonymous substitution rate at a specific locus per $N_{e}$ generations (Amei and Sawyer 2012), and we divide the $\theta_{s}$ by the total number of protein coding synonymous sites at that locus to obtain a rate of substitution per synonymous site per $N_{e}$ generations, denoted by $\tilde{\theta_{s}}$. To estimate the haploid effective population size $N_{e}$ ($N_{e}/2$ for diploid population size) for each of the 10 rice groups, we used the average of the $\tilde{\theta_{s}}$ over the four functional regions as a final estimate of synonymous substitution rate and compared it with a neutral substitution rate of $10^{-8}$ per site per generation (Caicedo *et al.* 2007).

## Results

### Divergence time

**Figure 2:**
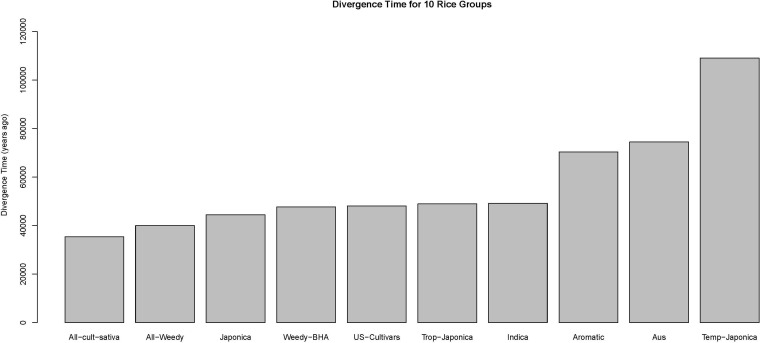
Bar graph of divergence times of 10 rice groups, *O. sativa*, from youngest to oldest: all cultivated sativa, all weedy rice, japonica, weedy rice BHA, US cultivars, tropical japonica, indica, aromatic, aus, temperate japonica. The unit is years ago.

The PRF model applied in this study estimates species divergence time in units of $N_{e}$ generations ago. Assuming 1 year as one generation for rice species, the time of differentiation in the unit of 1 year ago is given by multiplying the model estimated $t_{div}$ by $N_{e}$. For the 10 rice groups, the model estimated divergence times along with their 95% credible intervals as well as the corresponding converted speciation times are listed in Table S1, and a bar graph of the speciation times in ascending order is displayed in Figure 2. Across the 10 rice groups, *temperate japonica* is the oldest group that diverged approximately 110,000 years ago from its wild-type ancestor *O. rufipogon*, and the youngest is the group of *Oryza sativa* with an estimated divergence time of approximately 35,000 years ago (Figure 2).

### Selective effects

**Figure 3:**
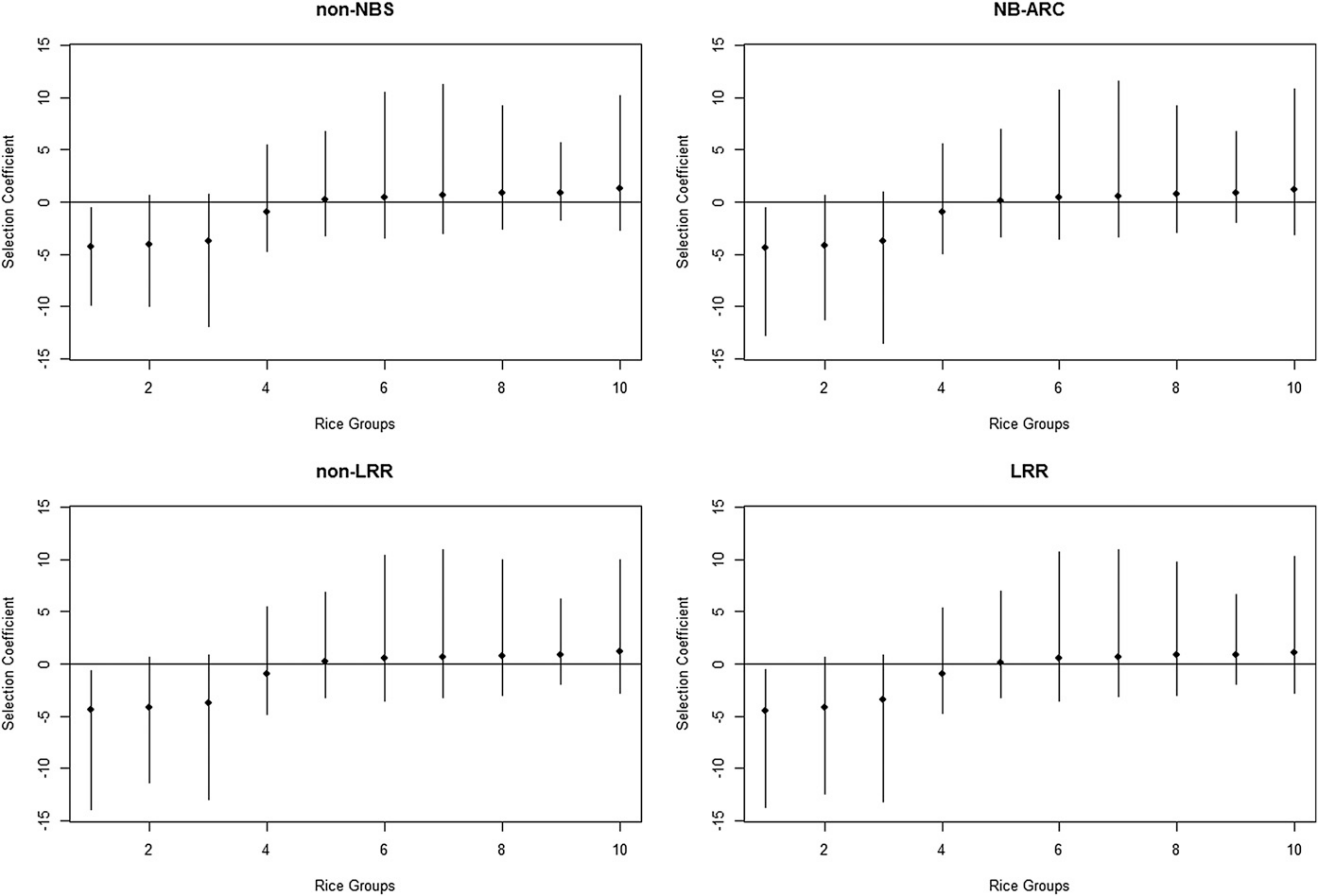
Selection coefficients (γ) for the four functional domains, non-nucleotide binding site (NBS), NB-ARC, non-leucine-rich repeat (LRR), and LRR with the 10 rice groups sorted by the values of the estimates (medians). Error bars represent 95% credible interval estimates of the γ.

Under the assumption that synonymous substitutions are neutral, the PRF model estimates selection coefficients *γ* of nonsynonymous substitutions for the four functional regions and the 10 rice groups. The sign of the*γ* indicates the direction of the selection in the sense that the substitution is beneficial if γ>0 and detrimental if γ<0. The absolute value of the *γ* measures the selection intensity of amino acid nonsynonymous substitution per functional region per $N_{e}$ generations. Based on our estimates, the selection coefficients of nonsynonymous substitutions at the four functional regions, NB-ARC, non-NBS, LRR, and non-LRR, are quite close to each other, and this result is consistent across the 10 rice groups. The medians and corresponding 95% credible interval estimates of the selection coefficients are given in Table S2 and plotted in Figure 3 with species sorted with the medians. Despite this, it is possible that some of these effects can be attributed to pooling groups that have been selected in different directions. Overall, there are four rice groups, *temperate japonica*, *aus*, *aromatic*, and *indica*, that have negative selection coefficients, and the *Pi-ta* gene is positively selected in the remaining six groups (Table S2). As far as the strength of the selection, the absolute value of the *γ* ranges from 1 to 4.31 for the four negatively selected groups and only 0.17 to 1.25 for the six positively selected groups (Table S2).

### Substitution rates

**Figure 4:**
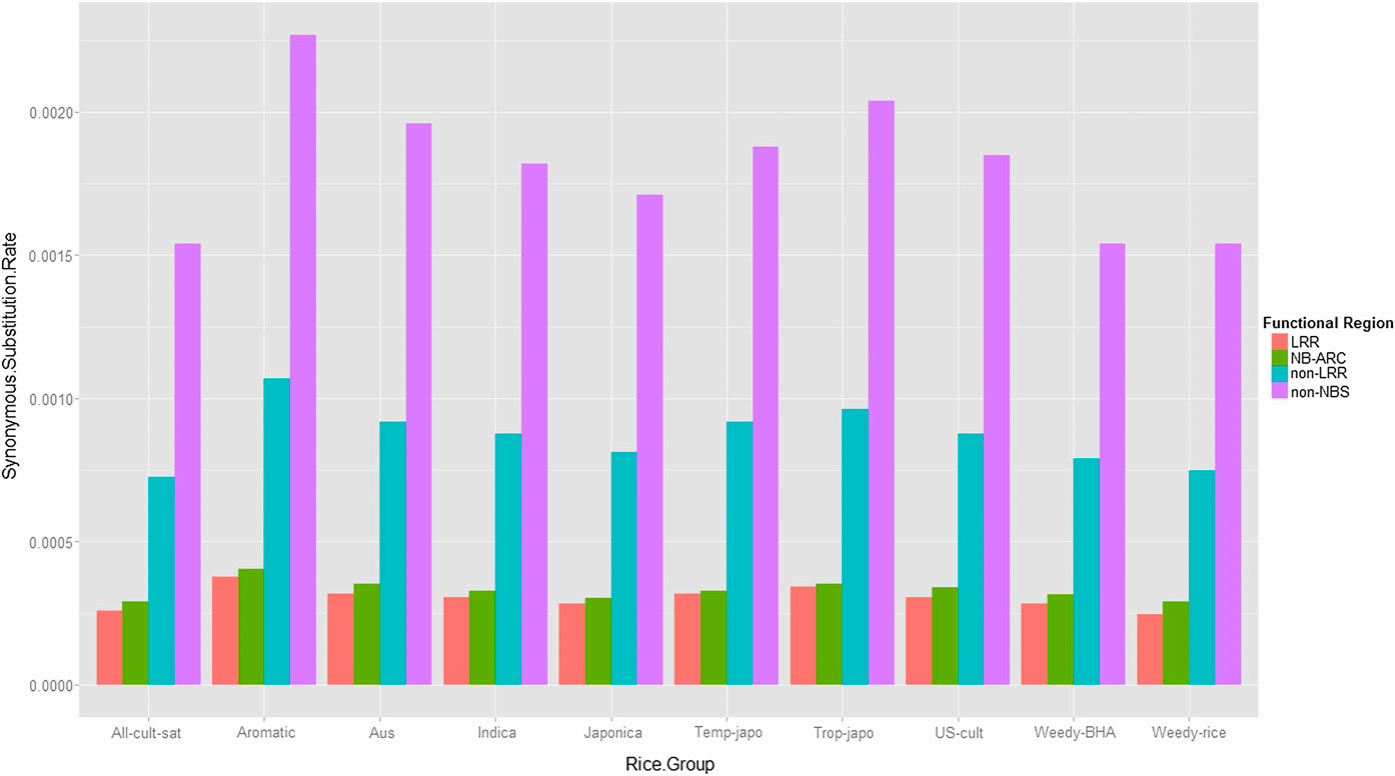
Synonymous substitution rates of the four regions across the 10 rice groups.

**Figure 5:**
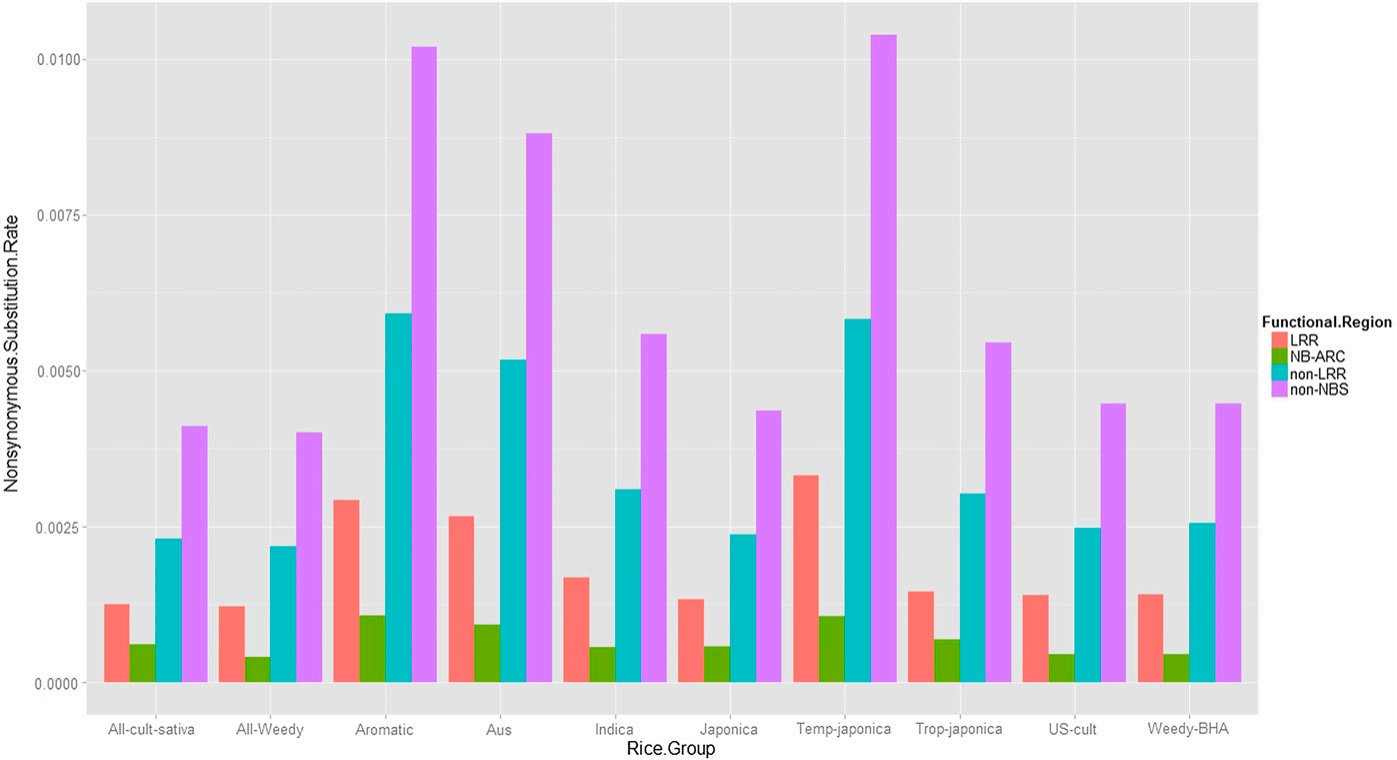
Nonsynonymous substitution rates of the four regions across the 10 rice groups.

As we mentioned previously, the substitution parameter *θ* ($\theta_{s}$ for synonymous sites and $\theta_{r}$ for non-synonymous sites) in the PRF model is an aggregate substitution rate, and we divide the *θ* by the total number of protein coding sites to obtain a site-specific rate. Numerical estimates of the synonymous and nonsynonymous substitution rates per site per$N_{e}$ generations of the four functional regions for the 10 rice groups are given in Tables 1 and 2. For both synonymous and nonsynonymous sites, substitution rates for noncritical domains are faster than those of critical domains (Figures 4 and 5). Specifically, the average synonymous substitution rates over the 10 groups are $1.82\times 10^{-3}$ in the non-NBS domain, $8.70\times 10^{-4}$ in the non-LRR domain, $3.31\times 10^{-4}$ in NB-ARC, and $3.04\times 10^{-4}$ in LRR. The average nonsynonymous substitution rates over the 10 rice groups are $6.19\times 10^{-3}$ in the non-NBS domain, $3.50\times 10^{-3}$ in the non-LRR domain, $1.87\times 10^{-3}$ in LRR, and $6.82\times 10^{-4}$ in NB-ARC. Our results showed that the nonsynonymous substitution rates are higher than the synonymous substitution rates.

**Table 1:** Estimate of synonymous substitution rate $\theta_{s}$, per site per *N*_*e*_ generations of *Pi-ta*

Rice group	Non-NBS	NB-ARC	Non-LRR	LRR
All cultivated *Oryza sativa*	$1.54\times 10^{-3}$	$2.90\times 10^{-4}$	$7.26\times 10^{-4}$	$2.59\times 10^{-4}$
Aromatic	$2.27\times 10^{-3}$	$4.04\times 10^{-4}$	$10.7\times 10^{-4}$	$3.77\times 10^{-4}$
Aus	$1.96\times 10^{-3}$	$3.54\times 10^{-4}$	$9.19\times 10^{-4}$	$3.18\times 10^{-4}$
Indica	$1.82\times 10^{-3}$	$3.28\times 10^{-4}$	$8.76\times 10^{-4}$	$3.06\times 10^{-4}$
Japonica	$1.71\times 10^{-3}$	$3.03\times 10^{-4}$	$8.12\times 10^{-4}$	$2.83\times 10^{-4}$
Temperate Japonica	$1.88\times 10^{-3}$	$3.28\times 10^{-4}$	$9.19\times 10^{-4}$	$3.18\times 10^{-4}$
Tropical Japonica	$2.04\times 10^{-3}$	$3.54\times 10^{-4}$	$9.62\times 10^{-4}$	$3.42\times 10^{-4}$
US cultivars	$1.85\times 10^{-3}$	$3.41\times 10^{-4}$	$8.76\times 10^{-4}$	$3.06\times 10^{-4}$
Weedy rice BHA	$1.54\times 10^{-3}$	$3.16\times 10^{-4}$	$7.91\times 10^{-4}$	$2.83\times 10^{-4}$
All weedy Rice	$1.54\times 10^{-3}$	$2.90\times 10^{-4}$	$7.48\times 10^{-4}$	$2.47\times 10^{-4}$
Average	$1.82\times 10^{-3}$	$3.31\times 10^{-4}$	$8.70\times 10^{-4}$	$3.04\times 10^{-4}$

NBS, nucleotide binding site; LRR, leucine-rich repeat.

**Table 2:** Estimate of nonsynonymous substitution rate $\theta_{r}$, per site per *N*_*e*_ generations of *Pi-ta*

Rice group	Non-NBS	NB-ARC	Non-LRR	LRR
All cultivated *Oryza sativa*	$4.12\times 10^{-3}$	$6.06\times 10^{-4}$	$2.31\times 10^{-3}$	$1.26\times 10^{-3}$
Aromatic	$10.2\times 10^{-3}$	$10.7\times 10^{-4}$	$5.92\times 10^{-3}$	$2.93\times 10^{-3}$
Aus	$8.81\times 10^{-3}$	$9.22\times 10^{-4}$	$5.17\times 10^{-3}$	$2.67\times 10^{-3}$
Indica	$5.59\times 10^{-3}$	$5.68\times 10^{-4}$	$3.10\times 10^{-3}$	$1.68\times 10^{-3}$
Japonica	$4.37\times 10^{-3}$	$5.81\times 10^{-4}$	$2.37\times 10^{-3}$	$1.33\times 10^{-3}$
Temperate Japonica	$10.4\times 10^{-3}$	$10.6\times 10^{-4}$	$5.83\times 10^{-3}$	$3.32\times 10^{-3}$
Tropical Japonica	$5.45\times 10^{-3}$	$6.94\times 10^{-4}$	$3.03\times 10^{-3}$	$1.46\times 10^{-3}$
US cultivars	$4.48\times 10^{-3}$	$4.55\times 10^{-4}$	$2.48\times 10^{-3}$	$1.40\times 10^{-3}$
Weedy rice BHA	$4.48\times 10^{-3}$	$4.55\times 10^{-4}$	$2.56\times 10^{-3}$	$1.41\times 10^{-3}$
All weedy Rice	$4.01\times 10^{-3}$	$4.04\times 10^{-4}$	$2.18\times 10^{-3}$	$1.22\times 10^{-3}$
Average	$6.19\times 10^{-3}$	$6.82\times 10^{-4}$	$3.50\times 10^{-3}$	$1.87\times 10^{-3}$

NBS, nucleotide binding site; LRR, leucine-rich repeat.

### Variation of *Pi-ta* haplotypes and phylogenetic relationships

**Figure 6:**
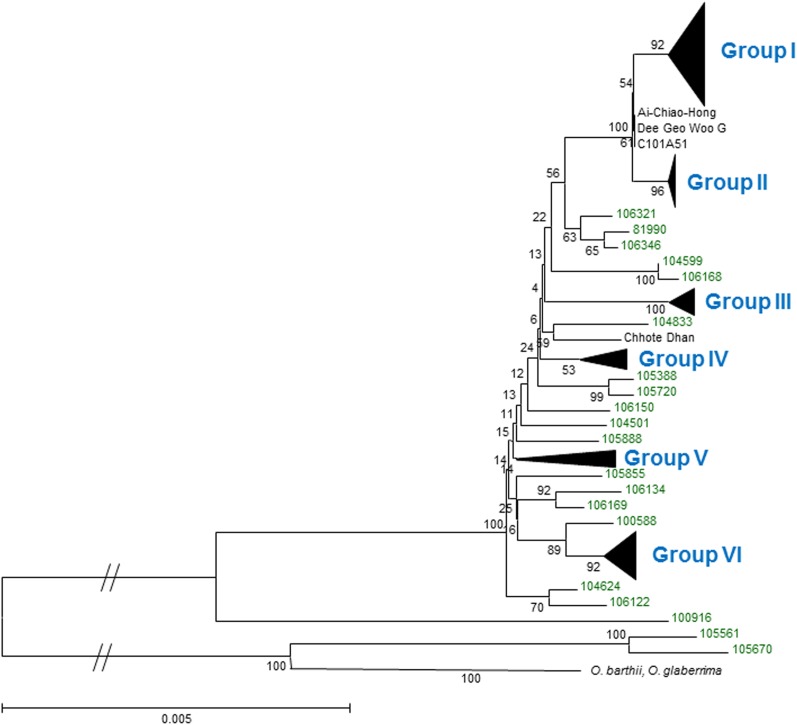
A phylogenetic tree describing the evolutionary relationship of the *Pi-ta* haplotypes among rice populations. The tree was constructed by the neighbor-joining method using MEGA 6 (http://www.megasoftware.net/). The phylogeny was tested by 500 bootstrap replications. The bootstrap values are shown on each branch of the tree, and the *O. rufipogon* accessions were marked in green.

A different number of the *Pi-ta* haplotype was determined in cultivated, weedy, and wild species rice (*O. rufipogon*), as shown in Table 3. The total number of haplotypes was 77 nucleotide and 48 in amino acid sequence of the *Pi-ta* coding region. The greatest number of haplotype was found in *O. rufipogon* (32 in nucleotide and 22 in amino acid), whereas 12 (nucleotide) and 7 (amino acid) were found in cultivated rice. The haplotype numbers were low in *temperate japonica* and *aromatic*. This may be due to the small number of samples used in this study (Table 3). As shown in Figure 6, we found a total of six major *Pi-ta* haplotype groups. All resistant rice accessions containing the resistant *Pi-ta* allele were found to belong to Group I, while all accessions possessing the susceptible *pi-ta* allele were in Group VI. Phylogenetic analyses revealed that the other *Pi-ta* haplotypes from cultivated (*aus*, *aromatic*, *indica*, and *japonica*) and weedy rice were present in Groups II, III, and IV. The accessions of *O. rufipogon* were found to be present in all *Pi-ta* haplotype groups. There was no cultivated or weedy rice found in Group V; only *O. rufipogon* was found. This finding suggests that the *Pi-ta* haplotype in this group may be a newly derived haplotype from *O. rufipogon* or present only in *O. rufipogon* (Figure 6).

**Table 3:** A number of *Pi-ta* haplotypes present in different groups of *Oryza sativa* or its wild species *O. rufipogon* used in this study

Rice Group	Number of Haplotypes (Nucleotide)	Number of Haplotypes (Amino Acid)
All cultivated *O. sativa*	12	7
Aromatic	1	1
Aus	4	2
Indica	7	3
Japonica	11	7
Temperate Japonica	1	0
Tropical Japonica	10	7
US cultivars	10	6
Weedy Rice BHA	7	3
Weedy Rice SH	0	0
All Weedy Rice	10	6
*O. rufipogon*	32	22
Total	77	48

The different *Pi-ta* haplotype was examined from the presence of polymorphic site at the full-length nucleotide sequence (2787 base pairs) of *Pi-ta*. The different *Pi-ta* haplotype was examined from the presence of polymorphic site at the full-length amino acid sequence (928 amino acids) of the Pi-ta protein.

## Discussion

*Pi-ta* is one of the best understood *R* genes for gene-for-gene resistance in plants (Bryan *et al.* 2000; Jia *et al.* 2003, 2009b). Several studies have been conducted in cultivated rice and its relative wild rice species to understand the molecular evolution of the *Pi-ta* gene (Caicedo *et al.* 2007 ; Huang *et al.* 2008; Lee *et al.* 2009, 2011; Molina *et al.* 2011; Thakur *et al.* 2013). Huang *et al.* (2008) and Thakur *et al.* (2013) analyzed the patterns of the *Pi-ta* gene evolution in cultivated and wild rice species by using statistical tests of neutrality such as Tajima’s D, Fu and Li’s D, Fay and Wu’s H, and the McDonald-Kreitman test. Based on their results, the null hypothesis of the neutral model was rejected by the values of Tajima’s D and Fu and Li’s D, but the Fay and Wu’s H and MK tests failed to provide evidence of selection. Using similar sets of statistics, Lee *et al.* (2009 and 2011) showed that the *Pi-ta* gene is under positive selection in cultivated rice and directional selection was observed in wild rice. The test statistics used in the existing studies are powerful in detecting selection when the underlying assumptions of the tests are met. However, they do not provide information on the magnitude and strength of the selection that they identified. On the other hand, the model applied in the current study, time-dependent PF model, is based on a quantitative theory for the amount of selection between two recently diverged species (Sawyer and Hartl 1992). Under certain assumptions, the model compares intraspecific polymorphism with interspecific divergence to generate quantitative estimates of genetic parameters such as selection coefficient, species divergence time and substitution rate. A recent application of the time-dependent PRF model to a nuclear and mitochondrial DNA dataset of 22 sister pairs of birds found evidence that selective effects in dry habitat species were stronger than those of humid habitat species (Amei and Smith 2014).

The rice group *temperate japonica* is shown to be the oldest group diverged from its wild-type ancestor *O. rufipogon* whereas the youngest is the group of *cultivated sativa* (Figure 2). Our estimates of species divergence times, in the unit of years ago, are much earlier than those in the existing studies. It has been reported that all forms of Asian rice, both *indica* and *japonica*, were domesticated around 8200 to 13,000 years ago although the precise date of the first domestication is still unknown (Caicedo *et al.* 2007; Molina *et al.* 2011). The differences are due to estimates of the haploid effective population size $N_{e}$ for various species. In PRF models, the speciation time $t_{div}$ is scaled in terms of the$N_{e}$. For example, we estimated that *indica* has diverged 0.27$N_{e}$ years ago, and this is in agreement with Caicedo *et al.*’s estimate of 0.21$N_{e}$ years ago. In their study, the time back to the beginning of domestication of 12,000 years was used as a standard and compared with their model estimated speciation time to estimate population size while we first estimated population size$N_{e}$ by using a neutral substitution rate of $10^{-8}$ per bp. However, one limitation of the PRF models is that they do not incorporate demographic features such as bottleneck, population subdivision and migration due to the model assumption of constant population size for both the ancestral species and its descendant.

As shown in Figure 3 (also Table S2), *Pi-ta* is under negative selection in *indica* while it shows positive selection in *japonica*, *tropical japonica*, and *weedy rice*. It has been reported that *Pi-ta* originated from landrace accessions and was used for many Asian and US cultivated rice varieties over the course of decades. In the US rice industry, *Pi-ta* has been used for many years to control rice blast, and most major US cultivars grown in Arkansas contain *Pi-ta* (Jia 2003, 2009; Jia *et al.* 2004b, 2009a). Thus, many accessions of Asian and US cultivated *japonica* rice used in this study possess the same *Pi-ta* allele that is resistant to common blast pathogen strains in the United States. This finding indicates that *Pi-ta* has been artificially selected during rice domestication in Asia and the United States, respectively. In addition, *Pi-ta* may have been evolved recently in *O. sativa*. As shown in Figure 6, the resistant *Pi-ta* haplotype present in *O. rufipogon*, which is the putative progenitor of cultivated rice, was found in many modern cultivated rice strains used in this study. Another interesting finding is that *Pi-ta* in US weedy rice is also under positive selection. The origin of the weedy species of rice in the United States is still not clear. It has been noted that weedy rice is genetically similar to wild species of rice, especially *O. rufipogon* (Gealy *et al.* 2003; Londo and Schaal 2007; Olsen *et al.* 2007). We found that US weedy rice has positive selection values, indicating that positive selection presents in US weedy rice. This result supports the previous report on the event of natural hybridization between cultivated and weedy rice (Chen *et al.* 2004; Gealy *et al.* 2003).

It has been known that LRR of *Pi-ta* is important to the function of blast disease resistance by recognizing the AVR-Pita effector. We also found that the amino acid replacement substitution rates are consistently low at LRR in all rice accessions. This result indicates that a strong positive selection has predominantly acted on the LRR regions of the *Pi-ta* gene, in agreement with previous studies (Huang *et al.* 2008; Lee *et al.* 2009; Thakur *et al.* 2013). Interestingly, in addition to the functional constraint of LRR, we found the lowest level of nonsynonymous substitution rates at the NBS-ARC domain of the *Pi-ta* gene in all rice accessions examined. It seems that the N-terminal coding region of the *Pi-ta* gene has played a critical role in the evolution of the *Pi-ta* function, similar to the role found in LRR. In our previous study (Lee *et al.* 2009), the resistance *Pi-ta* allele always contained a 3364-bp insertion near the approximate *Pi-ta* promoter region. It would be interesting to determine whether there is a correlation between the insert fragment and the evolutionary relationship of NBS-ARC at *Pi-ta*. In contrast to these two regions, we found that the 5′ regulatory sequence of the *Pi-ta* gene has the greatest level of nonsynonymous substitution rate among all rice accessions. The 5′ region of the gene has been known as a critical region for the meiotic recombination (Wu and Lichten 1994). Thus, the highly diverse region may suppress the recombination between the paralogs at the *Pi-ta* locus. The extreme divergence of the 5′ regulatory region was also identified in another rice blast *R* gene, *Pi2/9* locus (Zhou *et al.* 2007).

In conclusion, we demonstrated that the time-dependent PRF model presented in this study provides an analytical tool for understanding the evolution of disease *R* genes in crop plants. The model can be applied to multilocus datasets to quantitatively estimate selective effect, species divergence time and substitution intensity to yield a comprehensive view of genetic patterns across species. PRF models assume that nucleotide sites evolve independently, and, hence, they are more suitable to studies of genetic regions with high recombination rates. Simulation studies using multi-locus data have shown that in estimation of selective effects, methods based on PRF are relatively robust against the violation of this assumption (Abel 2009; Boyko *et al.* 2008; Bustamante *et al.* 2001; Zhu and Bustamante 2005). One limitation of the PRF model due to its restrictive assumption of constant population size for ancestral and daughter populations makes the model fail to capture any demographic changes that have happened in the history of the species. A more realistic PRF model with changing population size within species is under development, and it would be interesting to apply the new model to rice or other plant *R* genes to make statistical inferences about their evolution. Resulting knowledge will be essential for developing more effective disease management strategies.

## Supplementary Material

Supporting Information
