# Supplementary material for: Statistical Inference of Selection and Divergence of the Rice Blast Resistance Gene Pi-ta
Source: G3 (Bethesda). 2014 Oct 21;4(12):2425–32. doi: 10.1534/g3.114.014969 (PMC4267938; doi:10.1534/g3.114.014969)
Supplement: Supporting Information [file supp_4_12_2425__index.html]

Statistical Inference of Selection and Divergence of the Rice Blast Resistance Gene Pi-ta — Supporting Information 

# Statistical Inference of Selection and Divergence of the Rice Blast Resistance Gene *Pi-ta*

## Supporting Information for Amei *et al.*, 2014

**Files in this Data Supplement:**

- Supporting Information - Tables S1-S2 and File S1 (PDF, 147 KB)
- Table S1 - Median and 95% credible interval (CI) estimates of species divergence time *tdiv* (*Ne* generations ago), converted species divergence time *tdiv* (years ago) and haploid effective population size *Ne*. (PDF, 129 KB)
- Table S2 - Median and 95% credible interval (CI) estimates of selection coefficient γ, per *Ne* generations, over four functional regions for ten rice species. (PDF, 129 KB)
- File S1 - Rice accessions and supplemental resources for rice materials for this study. (.xls, 44 KB)
