# Supplementary material for: Statistical Inference of Selection and Divergence of the Rice Blast Resistance Gene Pi-ta
Source: G3 (Bethesda). 2014 Oct 21;4(12):2425–32. doi: 10.1534/g3.114.014969 (PMC4267938; doi:10.1534/g3.114.014969)
Supplement: Supporting Information [file supp_g3.114.014969_014969SI.pdf]

G3 · Genes | Genomes | Genetics

Statistical inference of selection and divergence of the rice blast resistance gene *Pi-ta*

Amei Amei*, Seonghee Lee[§], Kirankumar S. Mysore[§] and Yulin Jia[+1]

[*] Department of Mathematical Sciences, University of Nevada, Las Vegas, Nevada

[§]Plant Biology Division, The Samuel Roberts Noble Foundation, Ardmore, Oklahoma

[+] USDA-Agricultural Research Service, Dale Bumpers National Rice Research Center, Stuttgart, Arkansas

[1]Corresponding author: Yulin Jia, 1890 Hwy. 130 East, Stuttgart, AR 72160; Tel: 8706729300; Fax: 870673758. Email: Yulin.jia@ars.usda.gov

DOI: 10.1534/g3.114.014969

Table S1 Median and 95% credible interval (CI) estimates of species divergence time t_{div} (N_e generations ago), converted species divergence time t_{div} (years ago) and haploid effective population size N_e.

Rice sub-group	t_{div} with 95% CI	t_{div} (years ago)	N_e
All cultivated *Oryza sativa*	0.23 (0.11, 0.38)	35420	154000
Os Aromatic	0.31 (0.10, 0.60)	70370	227000
Os Aus	0.38 (0.16, 0.69)	74480	196000
Os Indica	0.27 (0.13, 0.43)	49140	182000
Os Japonica	0.26 (0.15, 0.42)	44460	171000
*Os*Temperate Japonica	0.58 (0.29, 1.22)	109040	188000
Os Tropical Japonica	0.24 (0.13, 0.39)	48960	204000
Os US Cultivars	0.26 (0.15, 0.40)	48100	185000
Os Weedy rice BHA	0.31 (0.17, 0.47)	47740	154000
All weedy Rice	0.26 (0.14, 0.41)	40040	154000

Source: These materials were used for a project supported by the National Science Foundation under grant no. 0638820 (Seonghee Lee, Stefano Costanzo,Yulin Jia, Kenneth M. Olsen, and Ana L. Caicedo. 2009. Evolutionary Dynamics of the Genomic Region Around the Blast Resistance Gene *Pi-ta* in AA Genome *Oryza* Species. Genetics 183:1315-1325).

Table S2 Median and 95% credible interval (CI) estimates of selection coefficient γ, per N_e generations, over four functional regions for ten rice species.

Rice sub group	Non-NBS	NB-ARC	Non-LRR	LRR
All cultivated *Oryza sativa*	0.45 (-3.4, 10.5)	0.43 (-3.5,10.7)	0.49 (-3.5,10.4)	0.50 (-3.5,10.7)
Os Aromatic	-3.73 (-11.9,0.8)	-3.73 (-13.5,1.0)	-3.79 (-13.0,0.9)	-3.38 (-13.2,0.9)
Os Aus	-4.04 (-10.0,0.6)	-4.13 (-11.2,0.6)	-4.19 (-11.3,0.6)	-4.14 (-12.4,0.6)
Os Indica	-1.0 (-4.7,5.5)	-0.95 (-4.9,5.6)	-1.0 (-4.8,5.5)	-1.0 (-4.7,5.4)
Os Japonica	0.88 (-1.7,5.7)	0.86 (-1.9,6.7)	0.87 (-1.9,6.2)	0.87 (-1.9,6.6)
OsTemperate Japonica	-4.31 (-9.9,-0.5)	-4.43 (-12.7,-0.5)	-4.43 (-13.9,-0.6)	-4.47 (-13.7,-0.5)
Os Tropical Japonica	0.64 (-3.0,11.2)	0.59 (-3.3,11.6)	0.63 (-3.2,10.9)	0.61 (-3.1,10.9)
Os US Cultivars	1.25 (-2.7,10.2)	1.14 (-3.1,10.8)	1.15 (-2.8,10.0)	1.12 (-2.8,10.3)
Os Weedy rice BHA	0.17 (-3.2,6.7)	0.16 (-3.3,7.0)	0.17 (-3.2,6.9)	0.16 (-3.2,7.0)
All weedy Rice	0.83 (-2.6,9.2)	0.80 (-2.9,9.2)	0.79 (-3.0,10.0)	0.82 (-3.0,9.7)

Source: These materials were used for a project supported by the National Science Foundation under grant no. 0638820 (Seonghee Lee, Stefano Costanzo,Yulin Jia, Kenneth M. Olsen, and Ana L. Caicedo. 2009. Evolutionary Dynamics of the Genomic Region Around the Blast Resistance Gene *Pi-ta* in AA Genome *Oryza* Species. Genetics 183:1315-1325).

File S1

Rice accessions and supplemental resources for rice materials for this study

Available for download as an Excel file at http://www.g3journal.org/lookup/suppl/doi:10.1534/g3.114.014969/-/DC1
