# Supplementary material for: Statistical Inference of Selection and Divergence of the Rice Blast Resistance Gene Pi-ta
Source: G3 (Bethesda). 2014 Oct 21;4(12):2425–32. doi: 10.1534/g3.114.014969 (PMC4267938; doi:10.1534/g3.114.014969)
Supplement: Supporting Information [file supp_g3.114.014969_TableS1.pdf]

Table S1 Median and 95% credible interval (CI) estimates of species divergence time t_{div} (N_e generations ago), converted species divergence time t_{div} (years ago) and haploid effective population size N_e.

**Rice sub-group**	**t_{div} with 95% CI**	**t_{div} (years ago)**	**N_e**
All cultivated *Oryza sativa*	0.23 (0.11, 0.38)	35420	154000
Os Aromatic	0.31 (0.10, 0.60)	70370	227000
Os Aus	0.38 (0.16, 0.69)	74480	196000
Os Indica	0.27 (0.13, 0.43)	49140	182000
Os Japonica	0.26 (0.15, 0.42)	44460	171000
*Os*Temperate Japonica	0.58 (0.29, 1.22)	109040	188000
Os Tropical Japonica	0.24 (0.13, 0.39)	48960	204000
Os US Cultivars	0.26 (0.15, 0.40)	48100	185000
Os Weedy rice BHA	0.31 (0.17, 0.47)	47740	154000
All weedy Rice	0.26 (0.14, 0.41)	40040	154000

Source: These materials were used for a project supported by the National Science Foundation under grant no. 0638820 (Seonghee Lee, Stefano Costanzo,Yulin Jia, Kenneth M. Olsen, and Ana L. Caicedo. 2009. Evolutionary Dynamics of the Genomic Region Around the Blast Resistance Gene *Pi-ta* in AA Genome *Oryza* Species. Genetics 183:1315-1325).
