# Supplementary material for: Statistical Inference of Selection and Divergence of the Rice Blast Resistance Gene Pi-ta
Source: G3 (Bethesda). 2014 Oct 21;4(12):2425–32. doi: 10.1534/g3.114.014969 (PMC4267938; doi:10.1534/g3.114.014969)
Supplement: Supporting Information [file supp_g3.114.014969_TableS2.pdf]

Table S2 Median and 95% credible interval (CI) estimates of selection coefficient γ, per N_e generations, over four functional regions for ten rice species.

Rice sub group	Non-NBS	NB-ARC	Non-LRR	LRR
All cultivated *Oryza sativa*	0.45 (-3.4, 10.5)	0.43 (-3.5,10.7)	0.49 (-3.5,10.4)	0.50 (-3.5,10.7)
Os Aromatic	-3.73 (-11.9,0.8)	-3.73 (-13.5,1.0)	-3.79 (-13.0,0.9)	-3.38 (-13.2,0.9)
Os Aus	-4.04 (-10.0,0.6)	-4.13 (-11.2,0.6)	-4.19 (-11.3,0.6)	-4.14 (-12.4,0.6)
Os Indica	-1.0 (-4.7,5.5)	-0.95 (-4.9,5.6)	-1.0 (-4.8,5.5)	-1.0 (-4.7,5.4)
Os Japonica	0.88 (-1.7,5.7)	0.86 (-1.9,6.7)	0.87 (-1.9,6.2)	0.87 (-1.9,6.6)
OsTemperate Japonica	-4.31 (-9.9,-0.5)	-4.43 (-12.7,-0.5)	-4.43 (-13.9,-0.6)	-4.47 (-13.7,-0.5)
Os Tropical Japonica	0.64 (-3.0,11.2)	0.59 (-3.3,11.6)	0.63 (-3.2,10.9)	0.61 (-3.1,10.9)
Os US Cultivars	1.25 (-2.7,10.2)	1.14 (-3.1,10.8)	1.15 (-2.8,10.0)	1.12 (-2.8,10.3)
Os Weedy rice BHA	0.17 (-3.2,6.7)	0.16 (-3.3,7.0)	0.17 (-3.2,6.9)	0.16 (-3.2,7.0)
All weedy Rice	0.83 (-2.6,9.2)	0.80 (-2.9,9.2)	0.79 (-3.0,10.0)	0.82 (-3.0,9.7)

Source: These materials were used for a project supported by the National Science Foundation under grant no. 0638820 (Seonghee Lee, Stefano Costanzo,Yulin Jia, Kenneth M. Olsen, and Ana L. Caicedo. 2009. Evolutionary Dynamics of the Genomic Region Around the Blast Resistance Gene *Pi-ta* in AA Genome *Oryza* Species. Genetics 183:1315-1325).
